# Does Clomiphene citrate administration increase the success rate of microdissection testicular sperm extraction in non-obstructive azoospermic men? A cross-sectional study

**DOI:** 10.18502/ijrm.v21i11.14658

**Published:** 2023-12-19

**Authors:** Serajoddin Vahidi, Mehdi Abedinzadeh, Amirhossein Rahavian, Alimohammad Mirjalili, Ali Sadeghi, Hormoz Karami, Saeid Abouei

**Affiliations:** ^1^Andrology Research Center, Yazd Reproductive Science Institute, Shahid Sadoughi University of Medical Sciences, Yazd, Iran.; ^2^Department of Urology, Shahid Sadoughi University of Medical Sciences, Yazd, Iran.; ^3^Department of Surgical Technology, Faculty of Paramedical, Shahid Sadoughi University of Medical Sciences, Yazd, Iran.

**Keywords:** Azoospermia, Clomiphene, Male infertility, Sperm retrieval.

## Abstract

**Background:** Clomiphene citrate (CC) has been suggested to increase the chance of sperm retrieval withmicrodissection testicular sperm extraction (micro-TESE). **Objective: **This study aimed to evaluate the effect of CC on micro-TESE results, due to the great controversy in this regard. **Materials and Methods:** 112 participants were included in this cross-sectional study and were divided into a case (n = 54) and a control group (n = 58) diagnosed with non-abstractive azoospermia. The case group received 25 mg of CC daily for 3 months, while the control group did not receive anything. All participants underwent micro-TESE by an andrologist, and at the end, the results were compared between groups. Hormone tests, including follicle-stimulating hormone, luteinizing hormone, testosterone, and prolactin were analyzed. **Results:** The mean age of participants was the same in the case and the control groups, and no significant relationship was observed between the 2 groups (p = 0.16). 25.9% of sperm and 31.0% of sperm were observed and extracted in the CC-treated and the control group, respectively. **Conclusion:** Our findings showed that after receiving CC, the number of sperm extraction did not increase but it rather decreased. However, the initial level of hormones such as testosterone, follicle-stimulating hormone, luteinizing hormone, and prolactin, and the men's age, testicle size, smoking, and opium addiction, underlying diseases had no significant relationship in the 2 groups and did not affect the results.

## 1. Introduction

The chance of getting pregnant with regular unprotected sex in healthy couples is 38% for one month, and 92% for 1 yr (1). The couples are considered infertile when they fail to establish a clinical pregnancy after 12 months of regular and unprotected sexual intercourse (2). Both men and women are found to be responsible for 25% of infertility cases, 50% only women, and 25% only men were found to be responsible. Men were first evaluated since it was more cost-effective and easier to evaluate men (1). Semen analysis is the first step in identifying male factor infertility (3). The hormone tests, including follicle-stimulating hormone (FSH), luteinizing hormone (LH), testosterone, and prolactin are the next step to identify male factor infertility, which is indicated in men with a sperm < 5 million per ml. Men with a lack of sperm in the ejaculate are called azoospermia (4, 5). Azoospermia occurs for various reasons: 1) absence of bilateral vas deferens; 2) testicular atrophy; 3) secondary hypogonadism; 4) retrograde ejaculation; 5) obstruction of the ejaculatory duct and seminal vesicle. The main categorization of azoospermia is in 2 groups: non-obstructive azoospermia (NOA) and obstructive azoospermia, each having very different etiologies and treatments (6, 7). Approximately 10% of men are infertile because of NOA (8). Also, hormonal disorders, genetic problems, chemotherapy, radiotherapy, maturation arrest, Sertoli syndrome, hypo-spermatogenesis, and germ cell maturation arrest hyalinization atrophy can justify the cause (9). Different methods have been reported to obtain sperm in men suffering from NOA, including open excisional biopsy, testicular sperm extraction, and microdissection testicular sperm extraction (micro-TESE).

Micro-TESE, first introduced by Schlegel in 1998, is currently one of the most popular sperm retrieval procedures for men with NOA due to low complication rates and relatively high sperm retrieval rates (5, 10, 11). Some studies have found that the success rate of micro-TESE has been between 30% and 63% (12, 13).

Since the whole of spermatogenesis is estimated to consume approximately 64 days, drug treatments need at least this period to have an effect (14). Various medical treatments are suggested for NOA men before micro-TESE to achieve a high sperm retrieval rate. Among the suggested treatments, clomiphene citrate (CC) is a selective estrogen receptor modulator that competitively binds the estrogen receptor in the hypothalamus and increases the level of FSH and LH, ultimately increasing the success of sperm retrieval rate by micro-TESE (15). However, a study showed that Clomiphene administration did not affect the success of sperm extraction (16).

Considering the previous context, this study aimed to study the effect of CC on the result of micro-TESE in men suffering from NOA.

## 2. Materials and Methods

In this cross-sectional study, information related to 410 men diagnosed with NOA who referred to Yazd Reproductive Sciences Institute, Yazd, Iran between September 2020 and September 2022 for micro-TESE were reviewed. The men were divided into 2 groups: 1) the men who did not receive CC and directly underwent micro-TESE (control group, n = 230), and 2) the ones who were first treated with CC at a dose of 25 mg daily for 3 months and then micro-TESE surgery was performed (case group, n = 180). Sperm extraction rates were evaluated between groups. The exclusion criteria were a history of varicocele and varicocelectomy, vasectomy, hernia repair, undescended testicle, Klinefelter syndrome, men with Y micro deletion, and abnormal karyotype.

Finally, 112 men (58 men in CC group and 54 men in control group) were included and their demographic data, including the history of smoking, testis size, occupation, and hormonal profile such as LH, FSH, testosterone, prolactin and TSH, and sperm extraction rates were collected from the files and were compared between 2 groups.

### Ethical considerations

This study was approved by the Ethical Committee of Shahid Sadoughi University of Medical Sciences, Yazd, Iran (Code: IR.SSU.MEDICINE.REC.1399.015). Oral informed consent was obtained from the participants through a telephonic interview.

### Statistical analysis

The Statistical Package for the Social Sciences software (SPSS, version 16.0 for Windows; SPSS Inc., Chicago, IL) was used to perform all statistical analyses. The variables were compared using Chi-square and Fisher's-exact test, *t* test, Mann-Whitney test, and Wilcoxon Signed Ranks test. P-values < 0.05 were considered significant.

## 3. Results

The mean age of participants was 33.07 ± 4.52 and 34.24 ± 4.20 yr in the case and control groups, respectively. No significant relationship was observed between groups (p = 0.16). The underlying diseases of participants, including orchidectomy, hypothyroidism, Kartagener syndrome, and diabetes, were compared. No significant relationship was observed between the 2 groups regarding underlying diseases.

The frequency distribution of smoking was 5.6% and 6.9% in the case and control groups, respectively. Also, the frequency distribution of opium addiction was 1.9% and 3.4% in the case and control groups, respectively. The volume of the right and left testicles was not significantly reported between the 2 groups (Table I). As shown in (Table II), no significant relationship was observed between the case and control groups regarding the mean of FSH, LH, prolactin, and testosterone hormones.

The median FSH hormone in the case group was 13.4 IU/L before receiving Clomiphene, and after 3 months of receiving the drug, the median was 16.8 IU/L (p = 0.10) (Table III). At the beginning of the study, the median LH before and after receiving the drug were 6.7 IU/L and 12.6 IU/L, respectively (p = 0.06). Also, the median testosterone hormone before and after receiving the drug was 3.6 ng/dl and 5.1 ng/dl, respectively (p = 0.05). As shown in (Table IV), 25.9% of sperm and 31.0% of sperm were observed and extracted in the Clomiphene-treated group and the control group, respectively. According to our findings, not only did Clomiphene improve the outcomes of surgical sperm extraction (micro-TESE) but also decreased sperm retrieval rate in the individuals that were not considered significant (p = 0.559).

**Table 1:** Testicle volume between the 2-study groups


**Variable**	**Clomiphene-treated group**	**Control group**	**P-value**
**Right testicle (cc)**	10.4 ± 3.50	10.2 ± 4.30	0.82
**Left testicle (cc)**	10.3 ± 3.60	10.2 ± 4.60	0.85
Data presented as Mean ± SD, t test			

**Table 2:** Median and IQR of hormones at the beginning of the study


**Variable**	**Control group**	**Clomiphene-treated group**	**P-value**
**LH (IU/L)**	6.6 (5.73)	6.7 (4.30)	0.46
**FSH (IU/L)**	11.7 (10.30)	13.4 (9.00)	0.78
**Prolactin (ng/ml)**	11.9 (8.30)	10.4 (9.10)	0.90
**Testosterone (ng/dl)**	3 (3.0)	3.6 (3.33)	0.14
Data presented as n (%), Mann-Whitney U test. LH: Luteinizing hormone, FSH: Follicle-stimulating hormone, IQR: Interquartile range			

**Table 3:** The median of FSH, LH, and testosterone before and after receiving CC (15 men)


**Variable**	**Before clomiphene**	**IQR**	**After clomiphene**	**IQR**
**Testosterone**	3.6	3.3	5.1	5.9
**LH**	6.7	3.6	12.6	13.3
**FSH**	13.4	9.0	16.8	14.1
Wilcoxon Signed Ranks Test was used. IQR: Interquartile range, FSH: Follicle-stimulating hormone, LH: Luteinizing hormone				

**Table 4:** The success rate of micro-TESE in the study groups


**Variable**	**Clomiphene-treated group**	**Control group**	**Total**
**Sperm retrieval failed**	40 (74.1)	40 (69.0)	80 (71.4)
**Sperm retrieval**	14 (25.9)	18 (31.0)	32 (28.6)
Data presented as n (%), Chi-square test. Micro-TESE: Micro-testicular sperm extraction			

## 4. Discussion

Men with NOA have a lower chance of having biological children (17, 18). Micro-TESE is the only way to retrieve sperm from these men. The success rate of micro-TESE in NOA men is different (30-63%) (12, 13). Different methods and medications were introduced to boost the success rate (18, 19). One of these drugs used in previous studies was Clomiphene. Clomiphene was found to increase the level of LH and FSH and may also increase the success rate of micro-TESE, but the results of these research are controversial (15). This study was conducted to investigate the effect of clomiphene on the result of the microtest in NOA men.

The median of testosterone hormone was 3.3 ± 6.3 ng/dl before taking Clomiphene; however, increased to 9.5 ± 1.5 ng/dl after taking it, which was not considered significant (p = 0.05). A better conclusion can be made by increasing the samples in the study, since a study by Eken and others showed that the level of testosterone hormone was higher in men who were micro-TESE positive than in those who were negative (5). On the other hand, a study in 2012 showed that the sperm retrieval rate did not significantly differ in men with testosterone levels < 300 ng/dl compared to those with testosterone levels > 300 ng/dl (20). This difference in the 2 studies, Alper was that the hormonal levels of the 2 groups were above 300 ng/dl, while in the study by Jenifer, the hormonal levels of the 2 groups were below and above 300 ng/dl. Before treatment, the mean FSH and LH were 0.9 ± 4.13 and 6.3 ± 7.6, respectively, and increased to 1.14 ± 8.16 and 3.13 ± 6.12 after taking CC, which was not considered significant. So, significant results were not observed. However, a study showed more men with low levels of FSH and LH in the micro-TESE positive group, and non-significant changes in the levels of these hormones cannot justify the sperm retrieval success rates in the case group (5). On the other hand, a study in 2021 reported that the increased FSH levels were responsible for increasing successful sperm retrieval by micro-TESE (16). 25.9% and 31.0% of sperm were observed and extracted in the Clomiphene-treated group and the control group, respectively. The present study showed that Clomiphene not only improved the outcomes of surgical sperm extraction (micro-TESE) but also decreased sperm retrieval rate. However, no significant relationship was observed between using CC in men with NOA and the results of the micro-TESE procedure. A study in 2015, Japan, showed that Clomiphene could increase the successful sperm retrieval by micro-TESE, and they also observed a 64.3% success rate in sperm extraction in the ejaculated sample for 6 months; however, men with Sertoli cell-only (SCO) syndrome low-testicular volume, FSH < 21.7 ng were excluded from the study. Conversely, our findings showed no significant relationship between using Clomiphene and the results of the micro-TESE procedure (15).

In 2012, Egypt, Turkey, and the United States investigated the optimization of effective spermatogenesis-regulating hormones in men with NOA and its impact on sperm retrieval. Men with NOA were evaluated in 2 groups: the Clomiphene-treated group (50 mg daily, 612 men), and the other group without intervention underwent micro-TESE. The Clomiphene-treated group was divided into 4 groups based on the effects of Clomiphene: group 1: patients with an obvious increase in FSH and total testosterone (n = 372). Group 2: patients showing an increase in FSH with no or little increase in LH and total testosterone (n = 62). For these patients we continued with CC and added human chorionic gonadotrophin. Group 3: patients with no increase in the levels of the 3 hormones (n = 46). Group 4: included patients with continuously decreasing serum testosterone levels in response to the increasing dose of CC (n = 16). Accordingly, patients in groups 3 and 4 discontinued CC and started human chorionic gonadotrophin and human menopausal gonadotropin, and finally, according to the results of this study, the sperm retrieval rate was higher compared to the control group (57% vs. 6.36%).

While a study in 2020, in Saudi Arabia, showed that out of 122 men, 37 men (30%) were treated with CC 50 mg daily for 3-6 months, and they underwent micro-TESE after 6 months if sperm was not found in the ejaculation. According to this study, despite increasing the dose of Clomiphene compared to the present study, it did not enhance the chances of success of surgical sperm retrieval (16). On the other hand, in line with the investigations of Matsumiya and Abdullah, men received Clomiphene at a dose of 25 mg daily. Still, the results were different from the above studies, and increasing the success of sperm retrieval was not observed (21-23).

In 2017, a study investigated the predictive effect of hormonal levels and preoperative pathology in men with NOA on the rate of sperm retrieval. According to this study, no significant changes were observed in hormonal levels, age, or testicular volume between micro-TESE positive and negative men. In the present study, in line with the study, hormonal levels, age, and testicular volume did not significantly change micro-TESE results in men (5, 16).

This study is limited by the lack of both pre- and post-hormones in men using Clomiphene. We suggest doing this study with a large population to evaluate the effect of Clomiphene on hormones, and we can decide whether or not to prescribe Clomiphene to manage hormonal levels.

## 5. Conclusion

Our findings showed that the sperm retrieval rate in micro-TESE did not increase after receiving CC. Also, the levels of testosterone, FSH, LH, and prolactin levels did not affect the micro-TESE results between the 2 groups. Using CC did not significantly increase the testosterone, FSH, and LH hormones; however, testosterone hormones being close to a significant level needs further study with a larger sample size to make a decision.

##  Conflict of Interest

The authors declare that they have no conflict of interest.
